# Pelvic Examinations by the Rectum

**Published:** 1876-03

**Authors:** G. A. Baxter

**Affiliations:** Chattanooga, Tennessee


### PELVIC EXAMINATIONS BY THE RECTUM.

By G. A. BAXTER, M. D., Chattanooga, Tennessee.

The tendency of late years has been towards boldness in investigation. The profession has not been satisfied with the meagre data upon which the last generation founded their knowledge, and have sought to open new fields, as well as more thoroughly to explore existing ones. The larygescope, the ophthalmoscope, the speculum, the uterine probe, the aspirator, all lay open new fields that were inaccessible to physicians but a comparatively short time since. They have all made wonderful changes towards conservatism, the great end of all surgical and medical treatment. We explore now the knee-joint, and extract its watery contents for investigation, obtaining a microscopical accuracy in diagnosis, where, but a few years back, the sight of such a limb would have caused the cutline to appear. We light, as with the sun, the hidden chambers of the larynx, the eye and the uterus, and operate in their darkened recesses with almost the certainty and success we could upon the hand or face. But it is not of these particularly that I wish to speak now. Still another field has been laid open to us within the last three or four years, showing the boldness of this tendency with greater force than either of the others mentioned. I refer to pelvic investigation, through the rectum. I stood, aghast, one afternoon, only two or three years since, at the Woman's Hospital in New York, when one of its distinguished surgeons informed his class that "he proposed now to try a method of investigation, lately ventured in England, to the diagnosis of what he believed to be a case of ovarian disease, by entering his hand and arm into the rectum as far as it became necessary." It was soon done. The sphincter ani was stretched and torn, the hand and arm entered two-thirds the way to the elbow. It returned, and he informed us that his diagnosis was now a certainty. An ovarian tumor existed about the size of a large orange. That it was perfectly unadherent. That, in addition, there were several—three, I think—fibroids attached around the base of the uterus; that this later organ was retroflexed, and its funders hypertrophied. I remember the rapidity of his detail and his ending words: "I have learned all this through the rectum." I saw this case afterwards operated upon and had an opportunity to confirm his diagnosis in every particular.

Before accepting any new discovery it becomes us to make thorough investigations of its merits for ourselves. We ask, then, each of himself—

1. In what does the operation consist?
2. What dangers arise therefrom?
3. What can be accomplished thereby?
4. In the light of the foregoing questions is the operation justifiable?

The operation is performed as follows: The patient is first anesthetized; then with the thumbs entered into the anus their dorsal surfaces approximating, dilitation is made, gradually at first, then with more force and rapidity, until the opening becomes large enough for the fingers and thumb to be forced inward in a crowded position. Dilitation is continued with these until the hand can enter, when further dilitation is unnecessary, as the arm now passes in with the ease that the body of the child follows the head in its passage through the soft parts outward. (Another method of dilitation is by means of air or water bags.) It is often necessary to rupture the sphincter-ani in part or thoroughly, and sometimes in a measure the perineum proper is torn and lacerated. The hand and arm are then entered and pressure made upward with one hand, while the operator presses downward and forward upon the abdomen immediately above the pubes, and the organs in the pelvis are examined separately and with thoroughness as to position, shape, size and attachments. The hand is then withdrawn, hemorrhage stopped, the laceration that has been caused, if any, sewed up at once, and the patient kept quiet until the parts are healed.

The objections to this operation are—

Its severe and painful character; its disagreeable nature and inaptitude for ordinary examination; the laceration that may take place; danger from hemorrhage; danger from septicuemia; the occasional production of rectal fistulas; inflammatory action, etc.

It cannot be denied by those who advocate the employment of this method of research that there is a certain amount of weight in each of these objections. It is indeed a painful, severe and to the operator as well as patient, a disagreeable procedure, bearing a close analogy, but more severe, generally, even, than fistula in ano or ruptured perineum in labor, and occasionally making both these operations imperative by the lacerations produced. But with the proper use of anesthetics these characteristics are in a great measure modified, if not entirely shunned. Pain ceases to be a consideration. The sphincter, external and internal, are relaxed, and become easy of dilitation, laceration is not so frequent, and with proper care and patience in dilitation, may, I think, generally be avoided Its disagreeable character should bear no weight at all (except in his bill) where duty is concerned in the mind of an intelligent and earnest physician.

Those who practice and advocate the operation do not claim for it adaptability to ordinary examination. They lay it down as a rule that it should *never* be performed in the office; that the same care is requisite for its after-treatment as should follow the operation for ruptured perineum, and that it is justifiable only in the event of doubtful diagnosis in determining the necessity or advisability of capital operations, as in the case of ovarian cists, and should in no case be employed where a simpler method of investigation will satisfy the mind of the physician, or preceding an operation when its necessity has been determined upon to ascertain more accurately its character, if it be a tumor, and adhesions, in order to facilitate the operation and lessen the danger arising therefrom. With these precautions it may safely be used by the majority of practitioners. Its dangers are comparatively slight. The laceration that may take place will heal with the same celerity of a ruptured perineum in labor, and this, with intelligent after-treatment, we have been taught to regard as an accident of comparative trifling importance. There are no arteries of any size except the pubic, that are in any danger at all, and the danger to this lies only in an anomalous position. A simple recognition of it is all that is necessary. The hemorrhage from the capillary distribution may be quite severe, but I think may always be controlled with the ordinary means for this purpose, generally by torsion alone. The most annoying result is the occasional production of blind rectal fistulas, where laceration has taken place to any considerable extent, or when hard pressure has been continued for any great length of time upon the same spot in the rectum by inexperienced persons. But under the light of the instructions thrown upon this subject of late years by Emmett, Sims, Thomas, etc., of New York, the operation for their cure has become easy and almost certain where destruction of tissue has not proceeded to any great extent. There remains but one real danger, then, and this is a result rather of the laceration that *may* take place, than of the operation itself. I refer to *septicæmia*. In hospital practice, in large cities, overcrowded, ill-ventilated, etc., this *is* a matter of no inconsiderable importance, but in smaller cities, and in private practice in the larger ones, where cases of septicæmia are so rare as to attract the curious, we can hardly give it much weight, and it should not offer a serious obstacle from its possible danger, when the stake at hand is a large one. We ask ourselves, then, what is to be gained by such a procedure? We answer broadly, that it is the key to a minute examination of every organ in the plevis. By it we can determine the size, shape, position, frequently the contents, of the uterus, fallopian tubes, overies, and in a measure, but with more difficulty, of the bladder; the attachments and adhesions of all; the possible existence of pelvic tumors, etc. Is not this sufficient? Does not this, in the light of the comparatively slight dangers, when operations such as overiotomy are under question, and even those of lesser severity, justify the procedure on the part of the physician and an endurance of the temporary discomfort on the part of the patient. My own opinion is that it does, and in all cases of doubtful diagnosis when operations are contemplated the procedure should be undertaken previous to the operation, and my judgment is founded on these two reasons, which I think embrace the whole case.

1. If an operation is determined upon by its accurate information the operator is enabled to act with more certainty and thereby increases his patient's chances for recovery.

2. It may demonstrate the utter hopelessness of recovery from a contemplated operation, thereby prolonging the existence of the patient for a variable period, and save a possible harm to the operator from the unfavorable termination.

I give this, however, only as my opinion, and invite a discussion by the profession on it.

